# Is Higher Viral Load in SARS-CoV-2 Associated with Death?

**DOI:** 10.4269/ajtmh.20-0954

**Published:** 2020-09-28

**Authors:** Klinger Soares Faíco-Filho, Victor Cabelho Passarelli, Nancy Bellei

**Affiliations:** Universidade Federal de São Paulo (UNIFESP), Laboratório de Virologia, Division of Infectious Diseases, Department of Medicine, Escola Paulista de Medicina (EPM), São Paulo, Brazil

## Abstract

There is no proven prognostic marker for patients hospitalized with COVID-19. We conducted a retrospective cohort study of patients hospitalized with COVID-19 from March 14, 2020 to June 17, 2020, at São Paulo Hospital, in São Paulo, Brazil. SARS-CoV-2 viral load was assessed using the cycle threshold (Ct) values obtained from a reverse transcription–PCR assay applied to the nasopharyngeal swab samples. The reactions were performed following the CDC U.S. protocol targeting the N1 and N2 sequences of the SARS-CoV-2 nucleoprotein gene and human ribonuclease P gene serving as an endogenous control. Disease severity and patient outcomes were compared. Among 875 patients, 50.1% (439/875) were categorized as having mild disease (nonhospitalized patients), 30.4% (266/875) moderate (hospitalized in the ward), and 19.5% (170/875) severe disease (admitted to the intensive care unit). A Ct value of < 25 (472/875) indicated a high viral load, which was independently associated with mortality (odds ratio [OR]: 2.93; 95% CI: 1.87–4.60; *P* < 0.0001). We concluded that admission SARS-CoV-2 viral load was independently associated with mortality among patients hospitalized with COVID-19.

## INTRODUCTION

The pandemic of SARS-CoV-2 has been responsible for causing more than 30 million infections around the world since December 2019.^1^ Although the severity is mild in most cases, several risk factors for moderate to severe COVID-19 have been identified, including older age and comorbidities.^2,3^ However, the association between SARS-CoV-2 viral load and clinical outcomes remains understudied. Two Chinese studies demonstrated that the viral load of hospitalized patients was higher than that of nonhospitalized ones.^4,5^ Also, a recent American study demonstrated that higher viral loads were associated with a higher risk of intubation and death for inpatients.^6^ Nevertheless, correlation between viral load and hospitalization risk for inpatients and outpatients remains unclear.

Detection of SARS-CoV-2 by the reverse transcription–PCR (RT-PCR) assay in nasopharyngeal swab specimens is the primary diagnostic method for COVID-19.^7^ Data concerning cycle threshold (Ct) values, which are inversely proportional to the amount of RNA virus’ copies, have been used as an inference of the viral load. Based on the hypothesis that the SARS-CoV-2 Ct value could also act as a biomarker of disease severity, clinical outcome, and mortality, we conducted a retrospective analysis of 875 patients diagnosed with COVID-19 at a large university hospital in São Paulo, Brazil, between March 2020 and June 2020.

## METHODS

### Study population and setting.

This retrospective observational cohort study involved patients and healthcare workers (HCW) who presented with respiratory symptoms from March 14, 2020 to June 17, 2020, at São Paulo Hospital in the Federal University of São Paulo, Brazil. All hospitalized patients considered suspected of COVID-19 had a nasopharyngeal swab sample collected on admission and analyzed for SARS-CoV-2. We also included all HCWs presenting with clinical suspicion of COVID-19 as part of a routine surveillance. All the specimens were stored in 2 mL sterile Ringer’s lactate solution (Fresenius, Bad Homburg vor der Höhe, Germany) in sterile tubes and transported to the Virology Laboratory at the Federal University of São Paulo for testing.

### Viral load analysis method.

We used the Ct values as a semi-quantitative measure of viral load. The amount of viral RNA copies present in the positive samples is inversely proportional to the corresponding Ct value. That is, the greater the amount of viral RNA, the lower the Ct value.

### Samples and RNA preparation.

Nasal swab samples were collected from the patients at admission. RNA of the samples was purified using the Quick-RNA Viral Kit (Zymo Research, Irvine, CA), according to the manufacturer’s instructions. The purified RNA was stored at −80°C.

### SARS-CoV-2 detection.

Viral detection was performed using the AgPath-ID One-Step RT-PCR reagents (ThermoFisher Scientific, Austin, TX), according to the manufacturer’s instructions. The reactions were performed in a 20-µL total reaction volume containing 5.0 µL purified RNA, 400 nM primers, and 200 nM probes following the CDC U.S. protocol targeting the N1 and N2 sequences of the SARS-CoV-2 nucleoprotein gene and human ribonuclease P gene serving as an endogenous control.^8^ For the semi-quantitative analysis, we used the Ct values of the more sensitive N2 target. Samples with Ct values < 40 were considered positive.

### Data collection.

Information on age, hospitalization status, and clinical outcome was retrieved from the electronic medical records of the patients. According to the WHO guidelines, patients were stratified into groups on the basis of age, as follows: 0–5, 5–14, 15–24, 25–34, 35–44, 45–54, 55–64, 65–74, 75–84, and > 85 years. Disease severity and clinical outcome were classified as follows: mild (no hospitalization), moderate (hospitalization in the ward for observation and oxygen therapy), severe (hospitalization in the intensive care unit), and discharge or death. Patients who were still hospitalized, awaiting a defined outcome, during data collection were not considered for the statistical analysis.

### Statistical analysis.

Statistical analyses were performed using the Student’s *t*-test for parametric data and the Mann–Whitney test for nonparametric data. Significance level was set at a *P*-value of < 0.05.

## RESULTS

Among the 875 individuals with laboratory-confirmed COVID-19, more than a half (50.9%, 446/875) were female patients. The median age was 48 years (range, 2–97 years), and the median Ct value was 24. A Ct value of < 25 (472/875) indicated high viral load and > 24 (403/875) low viral load.

**Figure 1.**
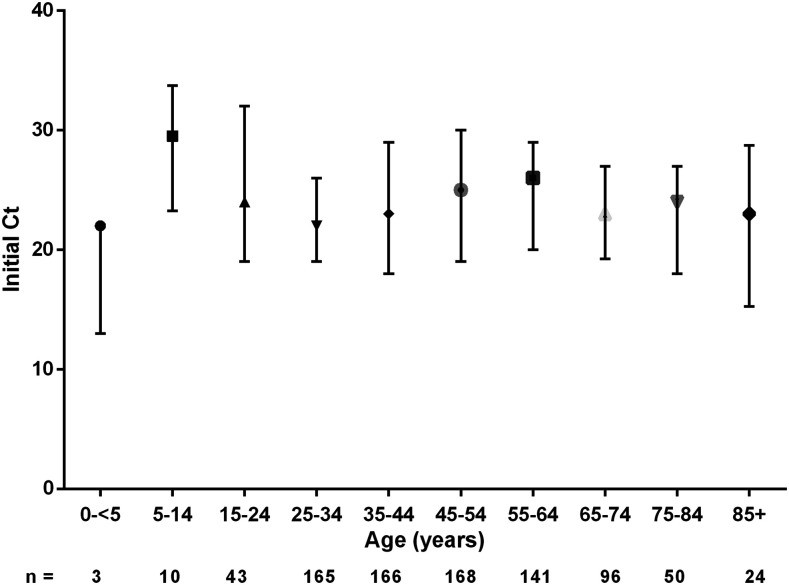
Initial cycle threshold (Ct) values from the swab samples in different age-groups.

The initial Ct values for each age-group are presented in Figure 1. We found no significant differences.

**Figure 2.**
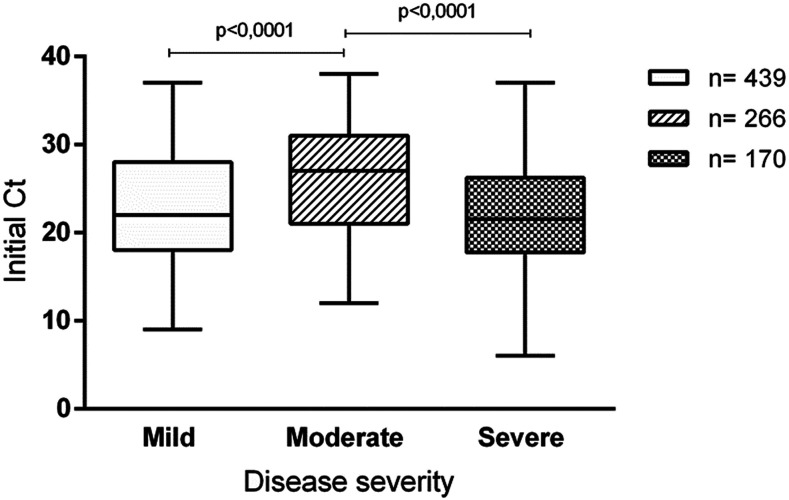
Initial cycle threshold (Ct) values from the swab samples obtained from patients with different clinical outcomes.

The Ct values were analyzed according to the disease severity—mild (50.1%; 439/875; median, 22); moderate (30.4%; 266/875; median, 27), and severe (19.5%; 170/875; median, 21.5)—in the 875 patients. The initial Ct value of patients with moderate disease was higher and significantly different from that of patients with mild (*P* < 0.0001) and severe (*P* < 0.0001) diseases (Figure 2).

**Figure 3.**
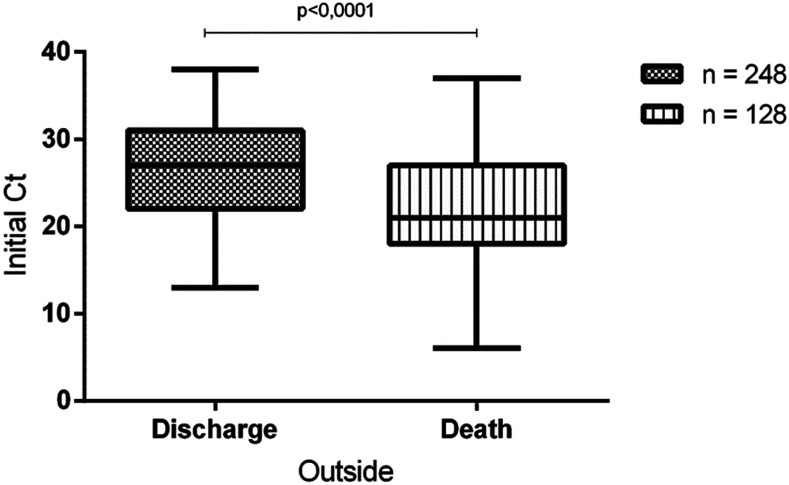
Cycle threshold (Ct) values from the swab samples according to discharge or death.

Comparison of the initial Ct value with clinical outcome (discharge or death) showed that survivors presented a significantly higher value than that of non-survivors: median Ct values were 27 and 21, respectively (*P* < 0.0001; Figure 3). Mortality rates were 46% (87/191) among patients with a high viral load (Ct < 25) and 22% (41/185) among patients with a low viral load. The risk of in-hospital mortality was also higher in patients with a high viral load (Ct < 25) than in those with a low viral load (Ct > 24), and this association was statistically significant (OR: 2.93; 95% CI: 1.87–4.60; *P* < 0.0001).

## DISCUSSION

In this study, a higher admission SARS-CoV-2 viral load (Ct values < 25) was associated with an outcome of death, as also demonstrated previously.^6^ The correlation of viral load with clinical outcome and disease severity has been investigated with different endemic respiratory viruses, but most results have not been conclusive.^9–12^ However, in the case of pre-pandemic avian influenza H5N1, higher viral loads were associated with more severe disease and poorer outcome,^13^ as seen with SARS-CoV-2 in our study.

Notably, SARS-CoV-2 viral load distribution according to the Ct values from initial samples followed a V curve in this study, with mildly affected and critically ill patients having higher viral loads than the hospitalized ones with a better outcome. First of all, the population with mild disease in this study mainly included healthcare workers tested in a very early stage of disease, when viral replication is very high.^4,5^ Furthermore, high viral loads in patients with mild disease were also described by Argyropoulos et al.^14^ However, although their study demonstrated that hospitalized patients had a lower viral load than nonhospitalized ones, disease severity was not categorized.

On the other hand, our study presented a unique stratified clinical expression of disease including mild (nonhospitalized), moderate, and severe disease in the hospitalized. So the difference in viral loads between moderately affected and critically ill patients might be explained by later viral peak and longer virus-shedding periods in the latter.^4,5^ This could also be a result of orientation to complete isolation at home if no warning signs were present, to avoid emergency rooms’ overcrowding. Therefore, most patients presented to the emergency department during a later period of disease, with more severe symptoms’ onset.

The results of this study must be interpreted considering the methodological limitations. We focused on virological aspects and did not include the effects of comorbidities, clinical symptoms, date of admission and sample collection, and use of antivirals or antibiotics because of the heterogeneity in the care of hospitalized patients, and requirement of a larger cohort for subgroup analysis. The duration of symptoms before testing may be an important variable. However, date of symptom onset was not consistently reported by medical assistants at this hospital. In any case, previous studies demonstrated that patients with other acute viral infections tend to present to the hospital after expected peak of viral load.^15^

In summary, admission SARS-CoV-2 viral load, demonstrated by the Ct value, was independently associated with mortality among patients hospitalized with COVID-19. These findings suggest that the Ct value could be used as a tool to help with the identification of patients at a higher risk for severe outcomes.
